# Selective Killing Effects of Cold Atmospheric Pressure Plasma with NO Induced Dysfunction of Epidermal Growth Factor Receptor in Oral Squamous Cell Carcinoma

**DOI:** 10.1371/journal.pone.0150279

**Published:** 2016-02-26

**Authors:** Jung-Hwan Lee, Ji-Yeon Om, Yong-Hee Kim, Kwang-Mahn Kim, Eun-Ha Choi, Kyoung-Nam Kim

**Affiliations:** 1 Department and Research Institute of Dental Biomaterials and Bioengineering, Brain Korea 21 PLUS project, Yonsei University College of Dentistry, 50–1 Yonsei-ro, Seodaemun-gu, Seoul, 120–752, Republic of Korea; 2 Institute of Tissue Regeneration Engineering (ITREN), Dankook University, Cheonan, Republic of Korea; 3 Plasma Bioscience Research Center, Kwangwoon University, 20 Kwangwoon-gil, Nowon-gu, Seoul, 139–701, Republic of Korea; University Paul Sabatier, FRANCE

## Abstract

The aim of this study is to investigate the effects of cold atmospheric pressure plasma (CAP)-induced radicals on the epidermal growth factor receptor (EGFR), which is overexpressed by oral squamous cell carcinoma, to determine the underlying mechanism of selective killing. CAP-induced highly reactive radicals were observed in both plasma plume and cell culture media. The selective killing effect was observed in oral squamous cell carcinoma compared with normal human gingival fibroblast. Degradation and dysfunction of EGFRs were observed only in the EGFR-overexpressing oral squamous cell carcinoma and not in the normal cell. Nitric oxide scavenger pretreatment in cell culture media before CAP treatment rescued above degradation and dysfunction of the EGFR as well as the killing effect in oral squamous cell carcinoma. CAP may be a promising cancer treatment method by inducing EGFR dysfunction in EGFR-overexpressing oral squamous cell carcinoma via nitric oxide radicals.

## Introduction

Cold atmospheric pressure plasma (CAP) is a promising therapy to selectively eradicate cancer cells [1,2]. CAP is a non-thermal plasma, which produces various plasma radicals at room temperature for medical applications [2]. CAP damages cancer cells intracellularly such as damaging deoxyribonucleic acid, mitochondria and causing apoptosis of the treated cells [3–5]. These changes are mostly explained by the accumulation of intracellular reactive oxygen species (ROS) in cancer cells, which induces mitochondrial dysfunction, sub-G1 phase arrest and deoxyribonucleic acid damage [6,7]. Compared to normal cells, cancer cells are more vulnerable to accumulation of ROS due to their elevated rates of ROS production, where they promote several aspects of proliferation and metastasis in cancer cells [8]. Taking advantage of the different response of cancer cells and normal cells to accumulated ROS, selective killing of CAP with respect to normal cells has been studied in ovarian, lung and metastatic breast cancer cells [9–11].

The CAP mechanism of action in cancer cells is mediated by high levels of ROS in media, alteration of cell cycle, changes in epigenetic regulation of cancer-relevant molecules such as p53, which regulates the cell cycle and functions as a tumor suppressor [12–14]. Highly reactive radicals and species from both exogenous and endogenous sources can readily damage essential cellular molecules, including cell cycle checkpoints, deoxyribonucleic acid and other organelles [15]. The cell cycle is vulnerable to targeting as it is made up of a series of synchronized events leading to duplication of cells and division producing two daughter cells.

To prevent oxidative stress induced damage to cell cycle and other biological process, all cells possess elaborate antioxidant defense systems that consist of antioxidants and detoxifying enzymes [16]. Among these, glutathione and other thiol-containing molecules, functioning as antioxidants with their thiol groups have been considered important cellular antioxidants due to relatively high concentrations in the cell [17]. Increased levels of thiols have been associated with increased tolerance to oxidant stresses and, consequently, measuring thiols is performed to quantitate the extent of cellular defense against oxidative stress [18–22].

Epidermal growth factor receptor (EGFR) plays a pivotal role in oral mucosal cells in regulating cell cycle, proliferation, differentiation and transformation [23], and it is commonly overexpressed in oral squamous cell carcinoma cells compared to normal oral mucosal cells [24]. The binding of epidermal growth factors activates EGFR, which as a membrane tyrosine kinase, in turn, initiates the signaling pathways for proliferation [25]. Oral squamous cell carcinoma cells with an abundance of EGFRs receive signals to proliferate uncontrollably, causing oral squamous cell carcinoma cells to divide unlimitedly [26]. When the EGFR kinase activity is targeted by therapeutic drugs, the oral squamous cell carcinoma cells lose proliferative ability, making these reagents effective in targeting oral squamous cell carcinoma cells [27,28].

Resistance to drugs limits the clinical benefit of EGFR-targeted therapies [29]. EGFR targeting drugs can also eventually alter the EGFR downstream signaling pathway dependence in the cancer cells and reduce drug effectiveness, which is a phenomenon called secondary resistance [30]. To overcome secondary drug resistance in EGFR-overexpressing cancer cells, the induction of radicals in oral squamous cell carcinoma cell with various methods has been studied. Singlet oxygen is one of radicals investigated, which rapidly disrupts the EGFR and its downstream signaling pathway [25]. Ultraviolet light has also been shown to downregulate the EGFR by causing conformational changes in the receptor and inducing apoptosis [31,32].

CAP also leads to singlet oxygen production, and it also generates various other reactive oxygen or nitrogen species as well. However, little is known about the influence of CAP on EGFR. For CAP-based therapies, if the interactions between EGFR and CAP-induced radicals lead to degradation and dysfunction of the EGFR, a combination of CAP treatment and conventional EGFR-targeted therapy is suggested as novel therapeutic means of preventing or overcoming secondary resistance in oral squamous cell carcinoma cells [33]. This combination-based therapy with CAP may potentiate EGFR-inhibitor targeted therapy in oral squamous cell carcinoma cells. As each class of the produced radicals has its own specific functions, determination of key radicals among CAP-induced radicals may be necessary in understanding CAP-induced selectivity in cancer cell therapy and future clinical applications [34,35].

The aim of this study was to investigate the selective killing effect of cold atmospheric pressure plasma with dysfunction of the EGFR in oral squamous cell carcinoma.

## Materials and Methods

### CAP device

**Fig 1:**
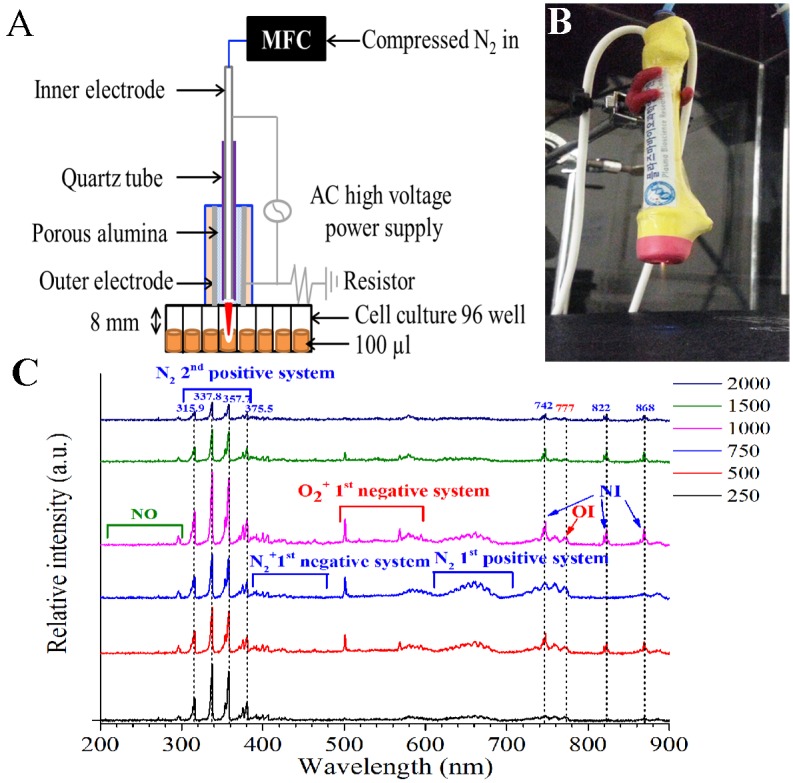
(A) Scheme of the CAP jet treatment on suspended cells in 100 μl media. (B) Images of CAP jet and induced plume. (C) Optical emission spectroscopy spectra.

CAP was developed and provided by Kwangwoon University (Fig 1A) [36]. Briefly, modulated CAP equipment is made up of an inner electrode made of tungsten with 1.2 mm depth and 0.2 mm thickness with 3.2 mm depth of quartz as a dielectric. The hole in the outer electrode made of stainless steel was 0.7 mm via the 2 mm height of porous alumina having a 150~200 μm pore size with 35% porosity. An oscilloscope (LeCroy Wave Runner 64XI 10 GS/s) (Teledyne LeCroy, Chestnut Ridge NY, USA), voltage probe (Tektronix P6015A High Voltage Probe, 1000:1) (Tektronix, Beaverton OR, USA) and Neon Trans (PNP-1000, max output voltage 15 kV, current 13 mA, 60 Hz in alternating current power) were implanted to detect output power in CAP equipment.

Discharge output voltage was set at 1.2 kV and its output power relating to intensity of CAP was calculated to be 0.51, 0.62, 1.98, 2.91, 2.4, 2.33W for 250, 500, 750, 1000, 1500 and 2000 standard cubic centimeters per minute (sccm) of flow rate, respectively, based on discharge power formula,

$$\frac{0.14}{T}\int_{0}^{T}v\left(t\right)\cdot i\left(t\right)dt$$

where *v(t)*, *i(t)*, *and T* are driving discharge voltage, current and voltage signal period with duty ratio (0.14) given by ratio of pulse on time (*t*_*on*_ = 25 ms) to summation of pulse on time and pulse off time (*t*_*on*_*+t*_*off*_ = 175 ms) in this experiment [37,38]. Plasma was formed by passing 250–2000 sccm of nitrogen flow rate through the CAP device. Control samples were not exposed to CAP. Each experiment underwent CAP treatment for 1 min with an 8-mm distance between the jet tip and the medium for plasma plume to touch media surface.

### Optical emission spectrum acquisition

In order to identify various excited plasma species produced in media from the application of CAP, the optical emission spectrum produced from CAP was collected by high-resolution fiber optic spectroscopy. From the spectrum, specific emission lines denote the presence of a specific radical or atom. The plasma plume of CAP was directed at the center of phosphate buffered saline solution surface, and the optical spectra was collected near the liquid medium at 5 mm away from the nozzle of outer electrode. The bombarded of phosphate buffered saline solution area was 2 mm in diameter and the plasma exposure time was set at approximately of 1 minute. The optical emission spectra were measured by a high-resolution CCD spectrometer (HR4000CG-UV-NIR, Ocean Optics, USA) coupled with an optical fiber. The instrument’s spectral range was from 200 to 1,100 nm. The optical emission spectrum was from the emitted light signals from the vicinity area ~ 2 mm in diameter in the central region of phosphate buffered saline solution surface to optical fiber whose diameter was 500 μm. The condition of optical emission spectra was set to be under atmospheric pressure with N_2_ gas flow rate Q of 50, 500, 750, 1000, 1500, and 2000 sccm, in this experiment. It is noted that the Reynold’s number (Re) [39] used for gas flow characteristics and plasma characteristics is approximated by Re = 820 x Q/ Rn for N_2_ gas where Q is flow rate in “lpm” and Rn is nozzle radius of gas flow in “mm.” The N_2_ gas flow rates under 250, 500, 750, and 1000 sccm were laminar flows as their Re values were less than 2000 (Re values of 410, 820, 1230, and 1640, respectively, in the experiment for the nozzle radius Rn of 0.5 mm) [39]. For gas flows of 1500 and 2000 sccm with Re values of 2460 and 3280, respectively, they would have flow turbulence as the laminar flow Re limit of 2000 is exceeded. With laminar N_2_ gas flows of under and including 1000 sccm, the plasma characteristics and compositions are uniform and there was no gas feedback for a turbulent flow to change the uniformity. The measurements were performed in triplicate, and representative results are shown.

### pH, temperature, composition and oxidation capacity after CAP treatment

To determine the interactions between CAP-induced radicals and the cell culture medium (100 μl) in each of 96 wells, the temperature, pH and oxidation capacity were measured. The changes in temperature and pH of the media or distilled water after CAP treatment (1 minute) with determined flow rate from 250 to 2000 sccm were measured using a digital pH-meter (Orion 4 Star, Thermo Scientific Pierce, IL, USA) and an indirect infrared thermometer (NC 100, Microlife, Switzerland). To determine the composition change in media after CAP treatment, Liquid chromatography—mass spectrometry was used. CAP treated or no-treated 100 μl of DMEM media was diluted one hundred times with distilled water containing 0.1% formic acid. Liquid samples were injected to liquid chromatography—mass spectrometry (Ultimate 3000 RS-LTQ Orbitrap XL, ThermoFisher scientific, USA). For LC, Aquity UPLC BEH C18 (2.1 x 100 mm, 1.8 um pore size) was used as column at 25°C. Flow rate was set at 0.3 ml/min and sample temperature was sustained at 20°C. For MS, the spray voltage was 5 kV. The flow-rate nitrogen sheath gas and the auxiliary gas were 50 and 5 (arbitrary units). The capillary voltage (V), tube-lens voltage (V), and capillary temperature (°C) were kept constant at 35 V, 120 V, and 275°C. The Orbitrap data were collected in the range of m/z 50–500. Resolution was determined as 60000. To measure CAP induced oxidation capacity in media, ferrous oxidation xylenol orange method (Quantitative peroxide assay kit, Thermo Scientific Pierce) was used according to the manufacturer’s protocol [40]. The Nitrate/Nitrite assay kit (Cayman Chemical, Ann Arbor, Michigan, USA) was performed to detect total nitrate/nitrite concentration in a simple, which are final products of NO in media according to the manufacturer’s protocol [41]. All the measurements were performed five times, and the averages with standard deviation are shown.

### Cell culture

A primary normal human gingival fibroblast-1 (HGF-1) cells (CRL-2014, ATCC, VA, USA) was used as a normal oral cell line at 5–10 passages [42]. HSC-2 (JCRB0622, JCRB Cell Bank, Japan) and SCC-15 (CRL-1623, American type culture collection) were chosen as oral squamous cell carcinoma cell lines. The cells (1 × 10^4^ cells/100 μl) were prepared from 90% confluent cultures biological experiments and seeded in each well of 96 wells (SPL, Pocheon, Gyeonggi-do, Korea). All biological investigations used 96 wells and employed the appropriate cell culture media (DMEM for HGF-1, DMEM/F-12(3:1) for HSC-2 and DMEM/F-12(1:1) for SCC-15) supplemented with 10% fetal bovine serum (Gibco, NY, USA) and 1% antibiotics (penicillin/streptomycin, Gibco). The cells were grown in an incubator at 37°C in a humidified atmosphere containing 5% CO_2_.

### Cell viability, apoptosis and cell cycle study

Cell viability was initially measured using a water-soluble tetrazolium salt assay (Ez-Cytox, Daeillab, Seoul, Korea). Following a 24-h incubation after CAP treatment (1 minute) using determined flow rate with various scavenger pretreatment (described below), a 10% water-soluble tetrazolium salt solution with supplemented media was added to each well, and the cells were incubated for 2 h to allow the formation of formazan crystals. One hundred μM of Trolox (6-hydroxy-2, 5, 7, 8-tetramethylchroman-2-carboxylic acid), carboxy-PTIO (C-PTIO) and uric acid treatment for 1h were used as scavenger for peroxyl radical (ROO-), nitric oxide (NO) and peroxynitrite anion (ONOO-) in media respectively. The signal absorbance was measured at 450 nm with a microplate spectrophotometer (BioTek, Winooski, VT, USA). Each condition was tested in sextuplicate. Cell apoptosis and necrosis was analyzed with the Annexin V-fluoroisothiocyanate apoptosis detection kit (#556570, BD Biosciences Pharmingen, CA, USA) following the manufacturer's instructions. To determine the cell cycle status, the cells were fixed with 4% w/v paraformaldehyde and resuspended in PI/RNase staining buffer (#550825, BD Biosciences) for 15 minutes at room temperature. The samples were analyzed by flow cytometry (FC 500, Beckman Coulter, CA, USA).

### Immunoblot assays

After CAP treatment (1 minute) using determined flow rate with various scavengers, clarified in below, was performed on cells, immunoblot assays were performed to observe protein expression. Before CAP treatment with 1500 sccm, 4 mM of N-acetyl-L-cysteine was used as a general scavenger of ROS and reactive nitrogen species (RNS) before 1500 sccm 100 μM of Trolox, C-PTIO and uric acid treatment for 1h were used as scavenger for peroxyl radical (ROO-), NO and peroxynitrite anion (ONOO-) in media respectively. Immunoblot assays were performed as described previously [43]. Cell lysates were collected using iced-cold cell lysis buffer (#9803, Cell signaling Technology, MA, USA) supplemented with protease inhibitor cocktail (Halt protease inhibitor, Thermo Scientific) and 1 mM phenylmethanesulfonyl fluoride. The cell lysates were resolved in sodium dodecyl sulfate-polyacrylamide gels and transferred onto nitrocellulose transfer membranes (Whatman, Germany) using a semi-dry transfer method (Trans-blot SD, Bio-Rad, CA, USA). The membranes were blocked in 5% non-fat milk (prepared in Tris-buffered saline supplemented with 0.1% Tween-20) at ambient temperature for 1 h. The membranes were then incubated with primary antibody at 4°C overnight. The following primary antibodies were used: EGFR (#2232), phospho-EGFR (#6963), and beta-actin (#4970). The membranes were washed for 1 h before incubation with horseradish peroxidas coupled secondary antibody (#7074). All antibodies were obtained from Cell Signaling Technology (MA, USA). The protein signals were visualized by enhanced chemiluminescence (Lumi-pico solution, Dogen, Korea) with exposure to X-ray film (AGFA, Belgian). Immunoblot experiments were performed in triplicate.

### Intracellular ROS detection

Cells in a 96-well plate were pretreated with or without C-PTIO and Trolox for 1 hr and stained with 1 mM 2’, 7’-dichlorofluorescein diacetate for a further 1 hr. The cells were then treated with CAP for 1 minute with various flow rates and incubated for 30 minutes. Cells were washed twice with phosphate buffered saline and reactive fluorescent units (excitation 480 nm, emission 530 nm) were detected with a fluorescence microplate reader (Varioskan Flash, Thermo-Fisher Scientific, Waltham MA, USA). The results were analyzed by first trypsinizing and detaching the cells and then normalizing the absorbance (intracellular ROS level) to cell number values determined by an automated cell counter (Luna, Logos, Anyang-Si, Gyunggi-Do, Korea). The equipment adjusted for cell plating differences among the wells.

### Thiol detection

To measure free thiol groups in cells untreated or CAP-treated for 1 minute, the cells were incubated with maleimide coupled to the fluorescent dye Alexa 633 (Molecular Probes, Thermo-Fisher Scientific). Thiol binding was measured by flow cytometry (Becton Dickinson, Durham NC, USA) according to a previously described procedure [20]. The thiol content in the lysed proteins was measured using a fluorescent probe in the Measure-IT^™^ Thiol Assay Kit (Molecular Probes, Thermo-Fisher Scientific). The fluorescence was recorded in a fluorometer at excitation/emission wavelengths of 494/517 nm (Varioskan Flash; Thermo-Fisher Scientific) [44]. The thiol content in each sample was calculated from a standard curve and normalized to the total protein, which was quantified by bicinchoninic acid assay protein assay (#23227, Thermo-Fisher Scientific).

## Results and Discussion

### Characterization of CAP-induced radicals

In this study, the quantity and type of CAP-induced radicals generated were analyzed by collecting optical emission spectrum off of the solutions treated by CAP (via high-resolution fiber optic spectroscopy (as detailed in the Materials and Methods). The CAP device was established under optimized conditions to maintain an overall plasma temperature close to room temperature (Fig 1B) [45]. In the wavelength range of 200–300 nm, there were few peaks regarding NO radicals. However, the dominant emission lines showed the presence of excited oxygen ions (O_2_^+^) at 500–600 nm and atomic oxygen (O I) at 777 nm (Fig 1C). We also detected reactive species associated with nitrogen and excited nitrogen molecules in the spectrum regions of 300–390 nm and 610–710 nm, respectively, as well as ionized nitrogen molecules (N_2_^+^) in the range of 390–480 nm. Atomic nitrogen (N I) was also found at 747, 822, and 868 nm wavelengths (Fig 1C). There was an increase in intensity of radical peaks with increasing flow rate until 1000 sccm flow rate and then the peaks diminished at the 1500 and 2000 sccm flow rates. The increase in the radical peaks with increasing flow rates until 1000 sccm was due to increased laminar flow increasing the electron collisions with the plasma gases resulting in more gas excitations. However, as shown in the experiment, for N_2_ gas flow rates at 1500 and 2000 sccm, the gases had turbulent flow and altered the plasma and the ROS or RNS emission characteristics resulting in the radical emission peaks diminishing. For the turbulent N_2_ gas flows, the gas excitations could not propagate a long enough distance for a proper CAP effect due to gas turbulent feedback. It is also noted that the NO peaks also decreased in the high gas flowing rates that were turbulent (Fig 1C).

### Characterization of CAP-treated media

Previous studies have reported that highly reactive radicals can effectively influence cells as they alter local concentration of composition, pH and temperature [46]. Liquid chromatography—mass spectrometry of CAP-treated cell culture media indicated changes in compositions of D-glucose and L-glutamine with a variation of ±10% compared to non-treated media. This was under the detection limit of liquid chromatography—mass spectrometry (S1 Table). There was also a decrease in the amount of phenol red in CAP-treated media, which could not have a biological effect [47]. Temperature changes after CAP treatment were detected under certain flow rates; however, the amount of change for a relatively short exposure time of 1 hr was not enough for significant cell killing by CAP treatment (S1 Fig) [48]. Significant pH change was also rarely detected (S2 Fig) [49].

**Fig 2:**
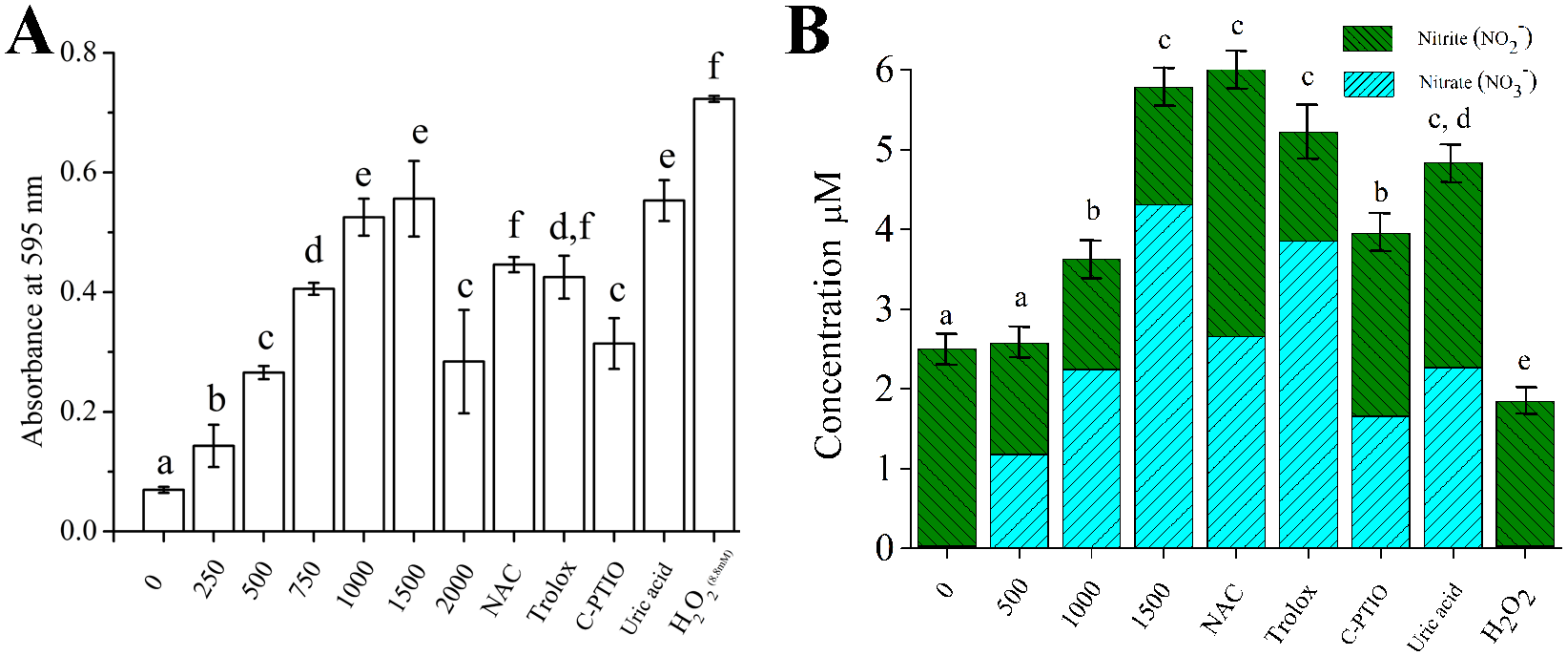
Oxidation capacity (A) and nitrite and nitrate formation (B) in cell culture media induced by CAP treatment. Different letters on the bar indicate significant differences among them (P<0.05). The assays (n = 5) were performed in triplicate, and representative results were shown with the average and standard deviation.

To investigate other effects of CAP-treated in culture media, the oxidation capacity of the media using the ferrous oxidation xylenol orange method with various radical scavengers treatments was examined [40]. An increase in oxidative conditions in cell culture media was observed depending on the flow rate (Fig 2A). The experiments were performed under 1500 sccm flow rates, allowing for laminar gas flow but at near maximal effects. Various radical scavenger treatments allowed for determining oxidative capacity changes with respect to certain radical groups. N-acetyl-L-cysteine at 4 mM was used as general scavenger for ROS and RNS species. Trolox, C-PTIO and uric acid each at 100 μM and for 1 hr of treatment were used as scavengers for peroxyl radical (ROO-), NO and peroxynitrite anion (ONOO-) species in media, respectively. When ROS reacts with aqueous media, peroxyl radicals (ROO-) are dominant in the culture media. When RNS reacts with aqueous media, NO and ONOO- are produced. According to the results, pretreatment with C-PTIO, scavenger of NO, diminished oxidation capacity of 1500 sccm CAP-treated media compared to N-acetyl-L-cysteine and Trolox (P<0.05). H_2_O_2_ at 8.8 mM was used as a positive control.

To study the RNS species interaction with cell culture media, total nitrate/nitrite concentration in the CAP-treated sample was detected by formation of nitrate/nitrite, which are the final products of RNS reacting with the media. Increases in levels of nitrogen radicals in cell culture media correlated with increases in flow rate (Fig 2B). N-acetyl-L-cysteine, Trolox, C-PTIO and uric acid were used to diminish nitrite and nitrate formation in CAP-treated media at 1500 sccm flow rate. Only C-PTIO, scavenger of NO, significantly reduced the rate of nitrite and nitrate formation (P<0.05). The H_2_O_2_ negative control could not induce RNS and, consequently, did not increase the concentration of nitrite and nitrate in the culture media (P>0.05).

### Selective killing induced by CAP treatment

**Fig 3:**
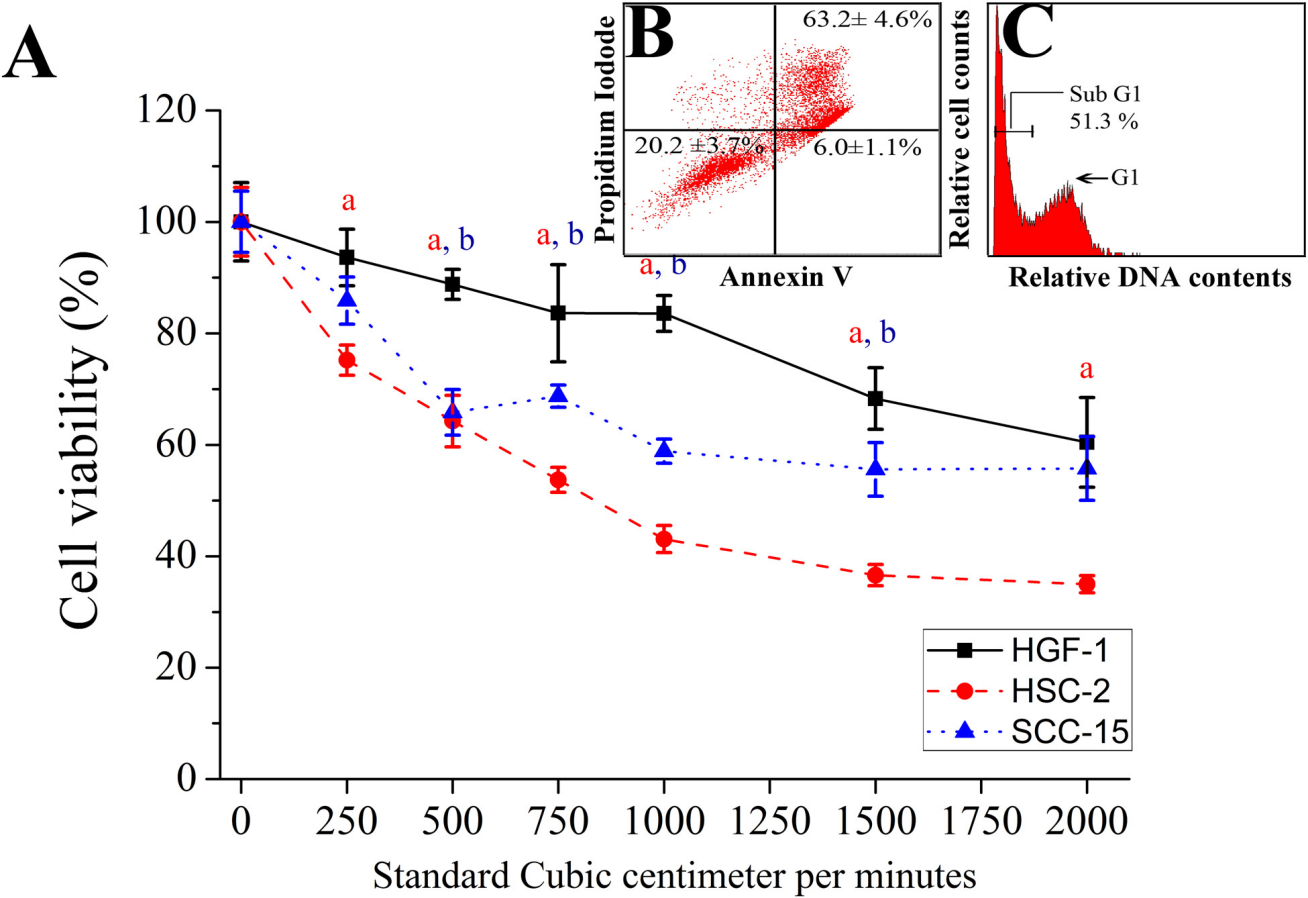
Selectivity of the CAP killing effect in three cell lines (normal cells [HGF-1] and two kinds of cancer cell lines [HSC-2 and SCC-15]) via sub-G1 arrest apoptosis. (A) Cell viability results from water-soluble tetrazolium assay after 24 h of CAP treatment (1 minute) with determined flow rate. (B) Annexin-PI staining and (C) cell cycle results were shown after 24 h of CAP treatment (1 minute) with 1500 sccm were performed in HSC-2. Red letter (a) and blue letter (b) indicate significant difference of cell viability in HGF-1 compared to HSC-2 and SCC-15 respectively (P<0.05). The assays (n = 5) were performed in triplicate, and representative results were shown with the average and standard deviation.

The selective cell killing brought about by CAP treatment was tested on three versions of suspended oral cells; this was investigated by the water-soluble tetrazolium salts viability assay and Annexin V-fluoroisothiocyanate apoptosis detection kit (Fig 3A and 3B). HGF-1 cells (CRL-2014, ATCC, Manassas, VA, USA) were used as a model of normal oral mucosa cells (used at 5–10 passages). These cells also had similar growth rates to the tested oral squamous cell carcinoma cells [42]. Oral keratinocyte were not chosen as our control normal cells; although they may be of closer origin to oral squamous cell carcinoma cells, the cell proliferative discrepancy of the keratinocytes allowed differences in cell viability in a 24 hr period potentially confounding any effects a CAP treatment might have. For the oral cancer cell lines, the oral squamous cell carcinoma cells HSC-2 and SCC-15 were chosen as test cell lines.

The water-soluble tetrazolium salt assay detects cell viability changes based on mitochondrial dehydrogenase activity, which is an important indicator of cellular metabolism. Upon CAP treatment, a significant decrease in cell viability was detected in oral squamous cell carcinoma HSC-2 and SCC-15 cells compared to control untransformed oral HGF-1 cells (P<0.05) (Fig 3A). The HSC-2 cells showed the most viability loss.

**Fig 4:**
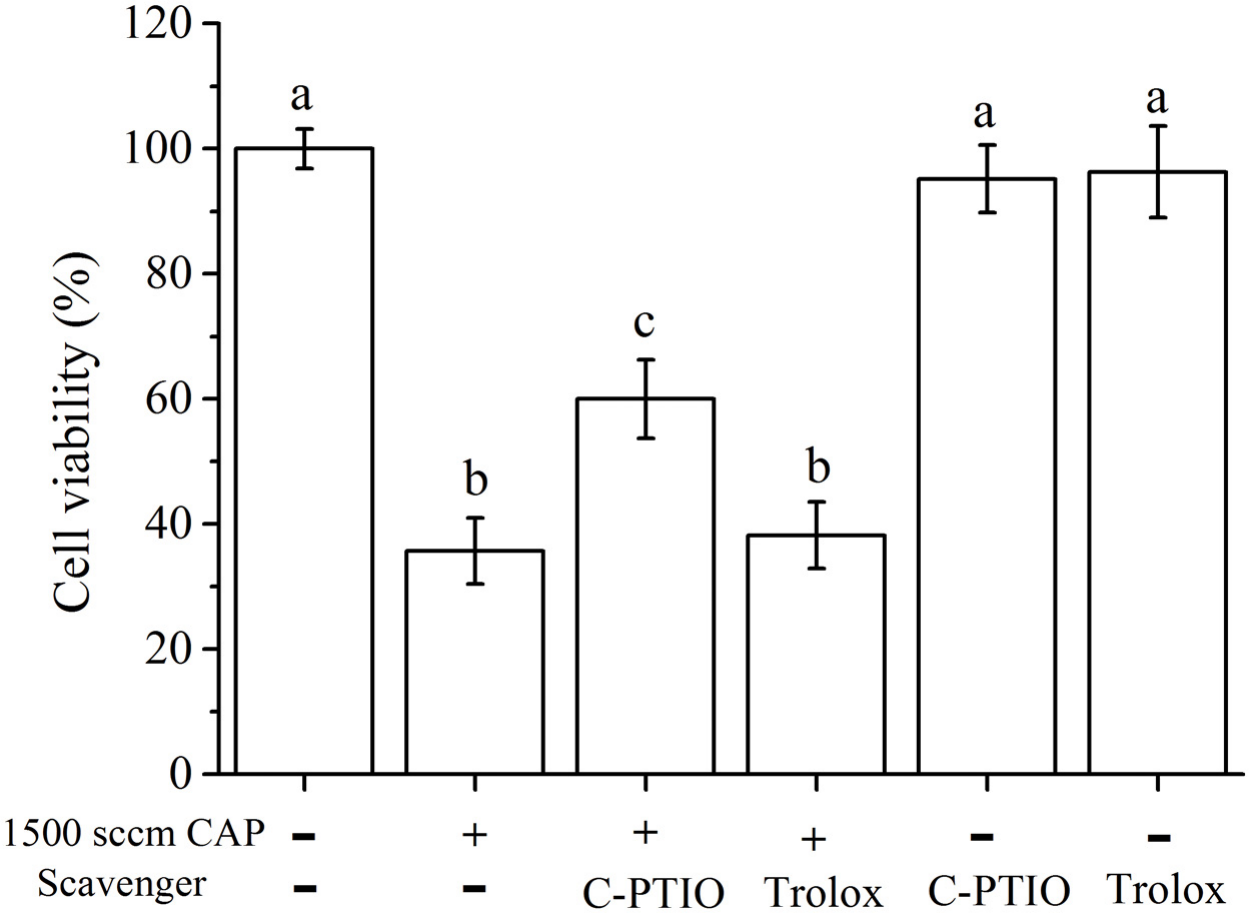
Cell viability of HSC-2 after CAP (1500 sccm, 1 minute) treatment after scavenger pretreatment. The assays (n = 5) were performed in triplicate, and representative results were shown with the average and standard deviation.

To evaluate whether the cell viability loss was due to induction of apoptosis, we ran the Annexin V-PI assay on the CAP-treated cells. With CAP-treatment, the number of Annexin V-PI stained cancer cells was increased, indicating an increase in apoptosis for the target cells. Compared with necrosis, inducing apoptosis in cancer cell is a more promising strategy for selective cancer treatment [50]. The Annexin V-PI staining confirmed selective cell targeting by CAP (S2 Table) as it led to more apoptotic death in cancer HSC-2 cells compared to the untransformed HGF-1 (Fig 3B). From the cell cycle analysis with PI, HSC-2 cells were killed via sub G1 arrest by CAP treatment with 1500 sccm flow rate, impacting deoxyribonucleic acid synthesis (Fig 3C) [3]. Contrary to HSC-2 cells, the HGF-1 cells did not show significant changes in cell cycle components before and after CAP treatment (S3 Fig). Treatment of HSC-2 cells with C-PTIO, the NO scavenger, before CAP treatment partially rescued the CAP killing effect (P<0.05, Fig 4) while the Trolox treatment had no impact on the cell killing (P>0.05, Fig 4).

### Role of EGFR in CAP treatment

**Fig 5:**
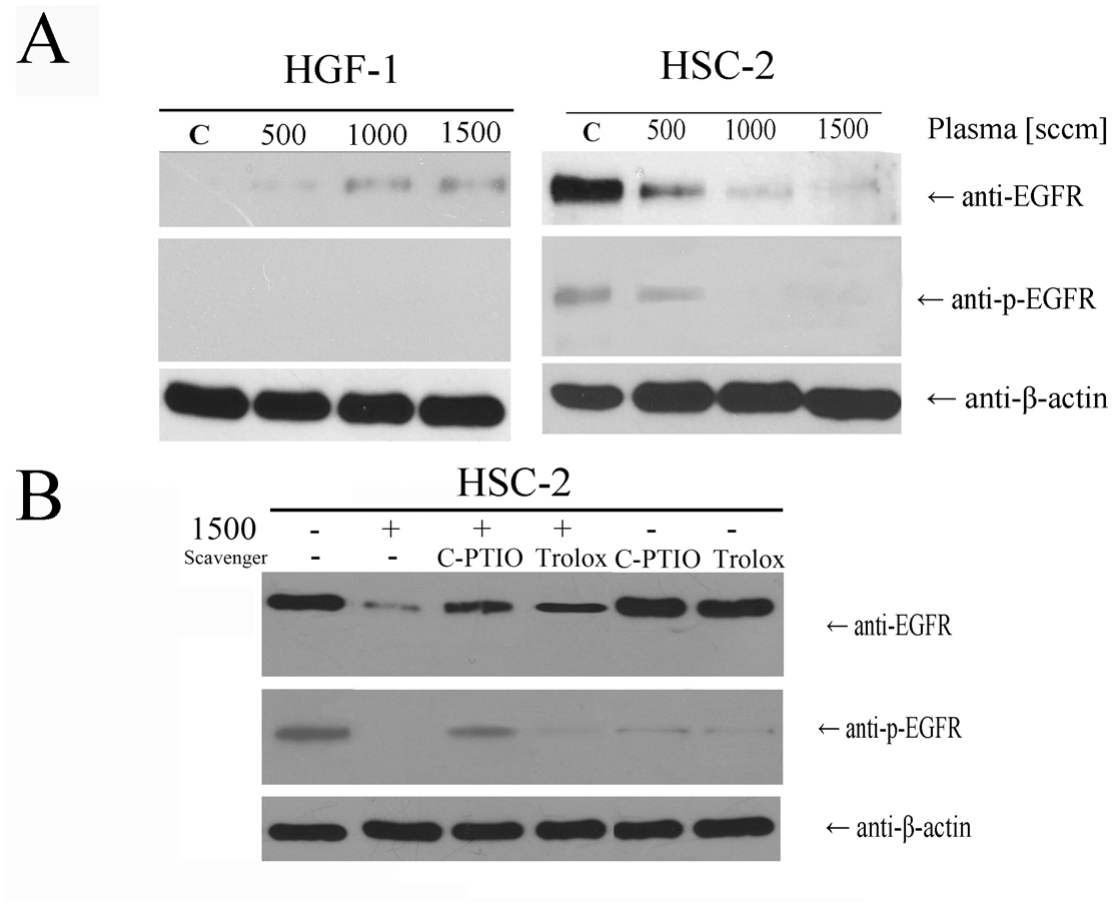
Immunoblotting assays for detecting EGFR, EGFR phosphorylation and housekeeping beta actin (A) after CAP treatment with further 24 hr further incubation. (B) Various kinds of radical scavenger pretreatment were performed before CAP treatment.

To explore the mechanism of how CAP treatment selectively targeted the oral squamous cell carcinoma cells, we determined the EGFR levels and the EGFR phosphorylation state at 24 hr after CAP treatment (Fig 5A and 5B). The degradation and dephosphorylation of EGFR in HSC-2 was related to the flow rate while, in HGF-1 cells, the expression of EGFR increased with flow rate but the EGFR phosphorylation was not changed (Fig 5A). EGFR is activated by specific ligands, namely EGFs, which results in activation of the EGFR pathway leading to cell surface loss and an increase in EGFR phosphorylation. In this study, we found that CAP treatment led to degradation and dephosphorylation of EGFR in HSC-2 cells, which suggested a deactivation of the EGFR pathway.

Targeting EGFR is a promising strategy for various tumor types utilizing the EGFR pathway. One question was whether the loss of EGFR was a secondary effect of cell apoptosis from the CAP treatment. Many investigators have observed that EGFR signaling cascades regulate proliferation and apoptosis of cells and, consequently, activation of EGFR is responsible for inhibiting apoptosis [50]. When EGFR inhibitors (i.e. cetuximab and gefitinib) are used to treat cancer cells, expression of both EGFR and phosphorylation of EGFR gradually decrease with increasing drug dose, as they induce an apoptosis of cancer cells [51,52]. There have been reports that cytotoxic drugs on tumor cells can have a synergistic killing effect on EGFR targeting as there is an increased or sustained level of EGFR expression on the cancer cells [53,54]. From previous published data and the observations presented in this work, CAP treatment allows the disruption of EGFR signaling in a specificity similar to that of biomolecules targeting EGFR directly and not with the general cytotoxic drugs.

EGFR dysfunction leads to cell apoptosis as seen with treatments like ROS and ultraviolet light for cancer or psoriasis. CAP produces various effectors such as ROS, RNS, electrons and ultraviolet light. Singlet oxygen has shown to inactivate the EGFR pathway [25]. However, H_2_O_2_ leads to the sustained activation of EGFR pathway [55]. These seemingly opposite effects of these two types of ROS sources may be explained by their different reactivities towards cellular molecules. H_2_O_2_ is mildly reactive and consequently exists long enough to diffuse through the cell. Singlet oxygen, on the other hand, is highly reactive with cellular molecules, and these include the protein components of membranes, but because of its short half-life, it reacts within distance of only ~100 nm [25]. Besides ROS, ultraviolet light produced can also downregulate EGFRs without phosphorylation changes and via conformational changes of EGFR [31].

To address the role of specific radicals for the CAP effect, various classes of radical scavengers were tested in whether they could alter and possibly reduce the CAP effect on target cells. The scavenger treated cells were tested for the CAP effect at 1500 sccm flow rate (Fig 5B). Degradation or dephosphorylation of EGFRs after CAP treatment was restored on cells pretreated with the NO scavenger, C-PTIO, or ROS scavenger, Trolox. C-PTIO pretreatment had a greater impact on rescue of EGFR membrane loss and EGFR phosphorylation than the Trolox pretreatment. According to the cell viability measures, C-PTIO treatment also partially rescued the CAP killing effect to HSC-2 but Trolox did not. Those data suggested that NO production had a major role in the inactivation of EGFR. Both C-PTIO and Trolox inhibited certain amounts of EGFR phosphorylation on cells not treated with CAP, which is seen as being due to the inhibition of oxidative stress involved in EGFR phosphorylation [56,57]. Given the above observations, further studies are required to elucidate whether NO radicals may induce down-modulation of EGFR in other cancer types as well.

**Fig 6:**
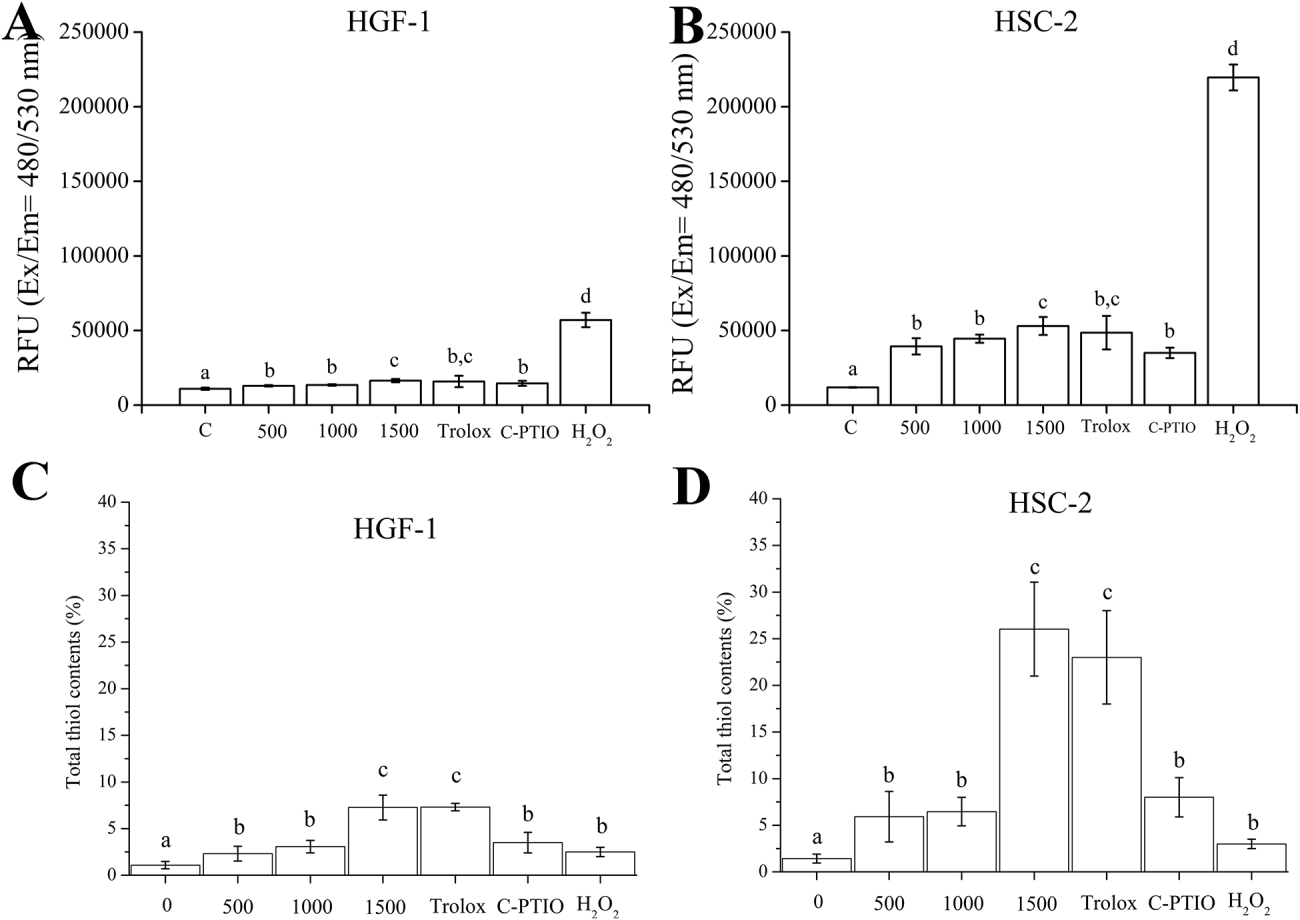
Intracellular reactive oxygen species (ROS) levels (A and B) and concentration of thiol contents representing antioxidant levels (C and D) after CAP treatment. Different letters on the bar indicate significant differences among them (P<0.05). The assays (n = 5) were performed in triplicate, and representative results were shown with the average and standard deviation.

The cellular thiol content, as an antioxidant functional group, as well as intracellular ROS levels may change with CAP-induced oxidative stress. Therefore, the intracellular ROS levels after CAP treatment were determined 1 hr after pretreatment with C-PTIO and Trolox. Free thiol content was also measured after CAP treatment for 24 hr after pretreatment with C-PTIO and Trolox [20]. Increases in intracellular ROS levels were seen along with increases in flow rate and this was detected in both HGF-1 and HSC-2 cells (Fig 6A and 6B). After pretreatment with the scavenger for NO, C-PTIO, the CAP mediated increase in ROS levels was significantly decreased for both HGF-1 and HSC-2 cells (P<0.05).

For cells treated by CAP, HSC-2 cells dramatically had higher thiol content while HGF-1 cells showed less of an increase in their thiol content (Fig 6C and 6D). For cells pretreated with C-PTIO, the increase of thiol content was significantly reduced (P<0.05) while Trolox pretreatment did not change the thiol contents for both cells treated with CAP (P>0.05). Interestingly, CAP treatment at 1500 sccm increased the thiol content more than H_2_O_2_ treatment, even though the H_2_O_2_ treatment produced more intracellular ROS.

Increased thiol content in cells may be interpreted as the cell compensating for oxidative stress. Theoretically, increased thiol content would be decreased by oxidation of thiol groups, which produce disulfide bonds and other oxidative-cysteine residues such as sulfinic acid, sulfonic acid and sulfonamide [58]. However, a sustained increase in thiol content was observed after CAP treatment.

**Fig 7:**
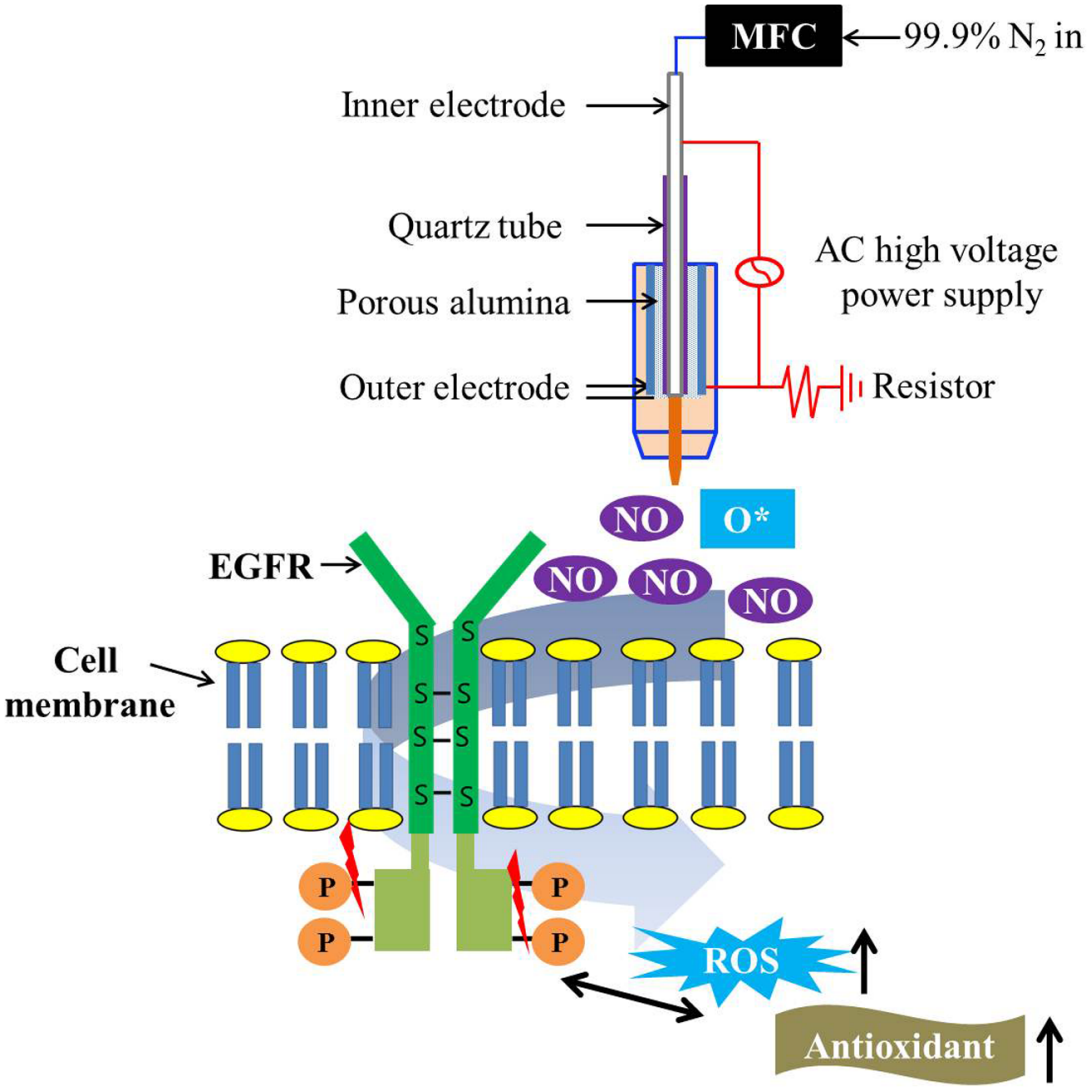
Schematic image of interaction between NO radicals from CAP and EGFR in EGFR-overexpressing oral squamous cell carcinoma cells. Dysfunction and degradation of EGFR were induced by an increase of intracellular ROS caused by NO radicals generated from CAP.

In terms of CAP induced factors influencing EGFR molecule, as a transmembrane receptor, EGFR contains a relatively high number of disulfide bonds and cysteine residues that interact with the inner and outer cellular environment [59]. The overexpression of thiol groups after CAP treatment could lead to an imbalance in the thiol-disulfide exchange of EGFR disulfide bonds and cysteine residues which can in turn lead to the degradation and dephosphorylation of the EGFR via formation of oxidative thiol residues. From the loss of EGFR pro-survival, anti-apoptosis signaling, the cancer cell may undergo apoptosis [19,60]. A schematic of the interaction between NO radicals from CAP and EGFR in EGFR-overexpressing oral squamous cell carcinoma cells is shown in Fig 7. Dysfunction and degradation of EGFR is brought on by an increase of intracellular ROS caused by NO radicals generated from CAP. Needless to say, further investigations are required to determine the mechanistic details involved in CAP-induced EGFR degradation and dephosphorylation.

It is noted in Figs 2A, 5, and 6 that the oxidative capacity of the CAP-treated media and the intracellular ROS as well as thiol contents is maximal at 1500 sccm flow rate, and not at 1000 sccm which had highest peak intensity values. It is conceivable that the ROS or RNS generation is interrupted at gas flow rates over 1000 sccm and Re because of gas turbulent feedback. The N_2_ gas flow rates under 1000 sccm were laminar flows as their Re values were less than 2000 (Re values of 410, 820, 1230, and 1640 for 250, 500, 750 and 1000 sccm, respectively) [39]. For gas flows of 1500 and 2000 sccm with Re values of 2460 and 3280, respectively, they would have flow turbulence, which were able to explain lower optical electronic spectrum value under 1500 and 2000 sccm compared to 1000 sccm counterpart. It is of note that both the profile of ions and ionized atoms in solution due to CAP as well as the intensity of plasma define the properties of the applied CAP [61] and its biological effect. However, there was significant increase of biological effect at 1500 sccm flow rate than 1000 sccm in this study. At 1500 sccm flow rate, the adherent cells were more easily affected by CAP because the volume of the media was less between adherent cells and surface of media which plasma encountered due to blow effect of gas flow. Because of the increased turbulence with increased gas flow, CAP could interact more with the media at 1500 sccm. Compared to 500 and 1000 sccm, 1500 sccm created more blow and turbulence in the media, helping CAP more efficiently interact with the adherent cells. This explained the significant decrease in cell viability and increase in oxidation capacity, nitrite and nitrate formation, increases in intracellular ROS, and thiol contents in 1500 sccm compared to 1000 sccm flow rates. For 2000 sccm flow rate, there would have been an overflow of media and detachment of cells due to an excessive blow effect [62]. It has been shown that there are no added gains for effects on cell viability for using a 2000 sccm setting rather than a 1500 sccm setting, and therefore, 1500 sccm was the upper flow limit used for the biological investigations in this study.

## Conclusion

This study documents the selective killing of oral squamous cell carcinoma cells via CAP-generated NO radicals. Degradation and dysfunction of EGFR were only observed in EGFR-overexpressing oral squamous cell carcinoma cells. Application of scavengers of NO to the cultures before CAP treatment rescued degradation and dysfunction of EGFR, as well as the killing effect of subsequent CAP treatment in oral squamous cell carcinoma cells. In summary, treatment with CAP may be a promising cancer treatment by targeting EGFR dysfunction in EGFR-overexpressing oral squamous cell carcinoma cells via generation of NO radicals in the applied solution. Future in vivo study using EGFR-null mutants will be needed to observe selective killing effects of CAP depending on the expression of EGFR.

## Supporting Information

S1 Fig Temperature of cell culture media after CAP or nitrogen only treatment according to the increase in flow rate. After CAP treatment was performed on 18°C cell culture media, temperature was measured. # showed significant difference compared to 18°C of no-treated cell culture media at a level of 0.05. ### showed significant difference at a level of 0.001.
(TIF)

S2 Fig pH of cell culture media (media) and distilled water (D.W.) after CAP treatment or nitrogen only treatment on each liquid according to the increase in flow rate. In both liquids, CAP treatment did not show significant difference compared to 1 h incubation media except 250 sccm of media and 1500 sccm of D.W. One # showed significant difference compared to 1 h incubated each solution at a level of 0.05. Three # showed significant difference at a level of 0.001.
(TIF)

S3 Fig Cell cycle analysis after CAP treatment with one normal human gingival fibroblast-1 (HGF-1) and two oral squamous cell carcinomas (HSC-2 and SCC-15). Sub-G1 arrest-related apoptosis was observed only in the HSC-2 cells and depended on the flow rate. (B) Percentage (%) of sub G1 through cell cycle analysis was shown. Assays were performed in triplicate, and representative data were shown.
(TIF)

S1 Table Component of media after CAP treatment (ppm). (XLSX)

S2 Table Results of Annexin V-PI staining after CAP treatment (%) (XLSX)
