# Supplementary figures and images for: Selective Killing Effects of Cold Atmospheric Pressure Plasma with NO Induced Dysfunction of Epidermal Growth Factor Receptor in Oral Squamous Cell Carcinoma
Source: PLoS One. 2016 Feb 26;11(2):e0150279. doi: 10.1371/journal.pone.0150279 (PMC4768860; doi:10.1371/journal.pone.0150279)

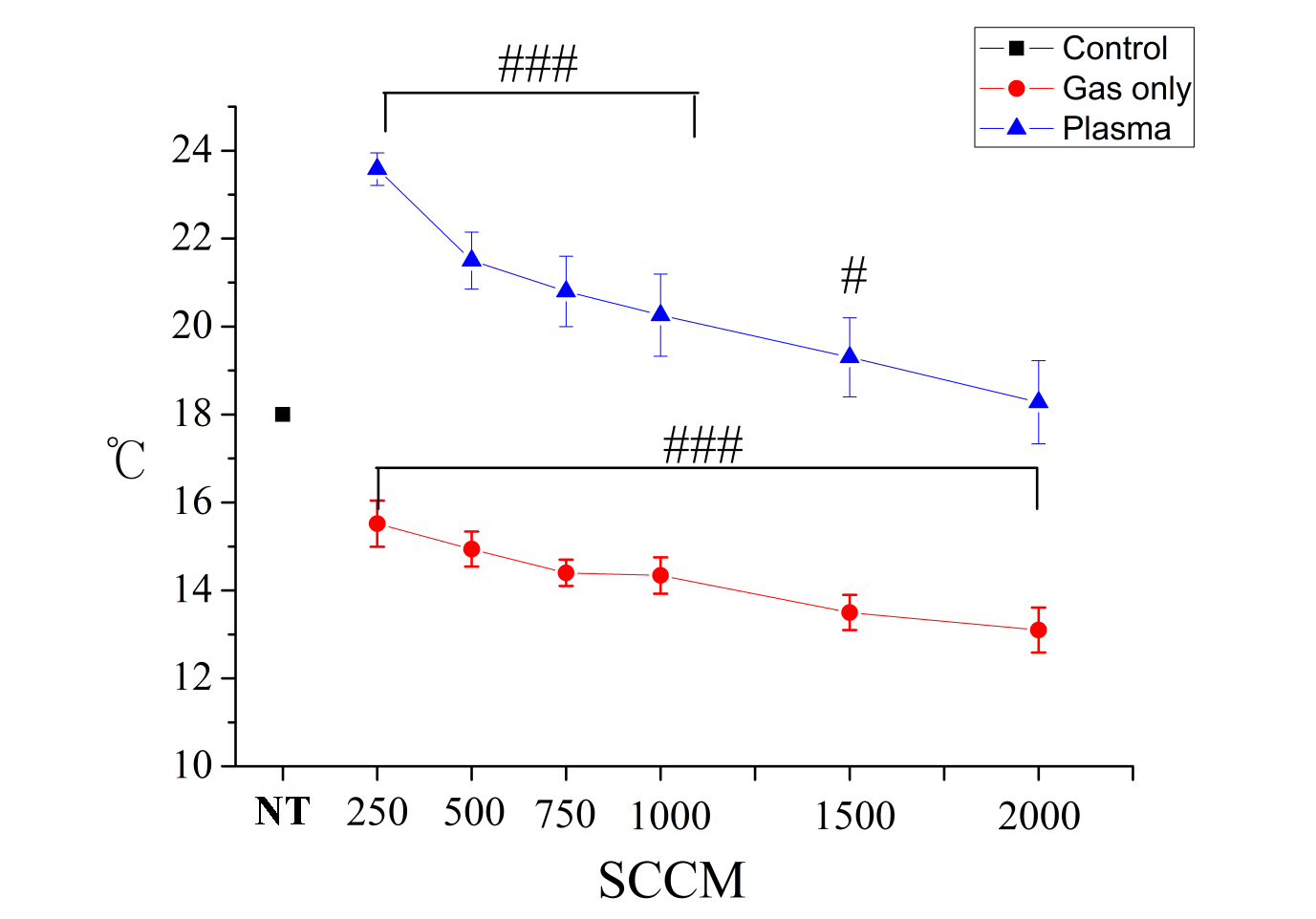

Supplement: S1 Fig — After CAP treatment was performed on 18°C cell culture media, temperature was measured. # showed significant difference compared to 18°C of no-treated cell culture media at a level of 0.05. ### showed significant difference at a level of 0.001. (TIF) [file pone.0150279.s001.tif]

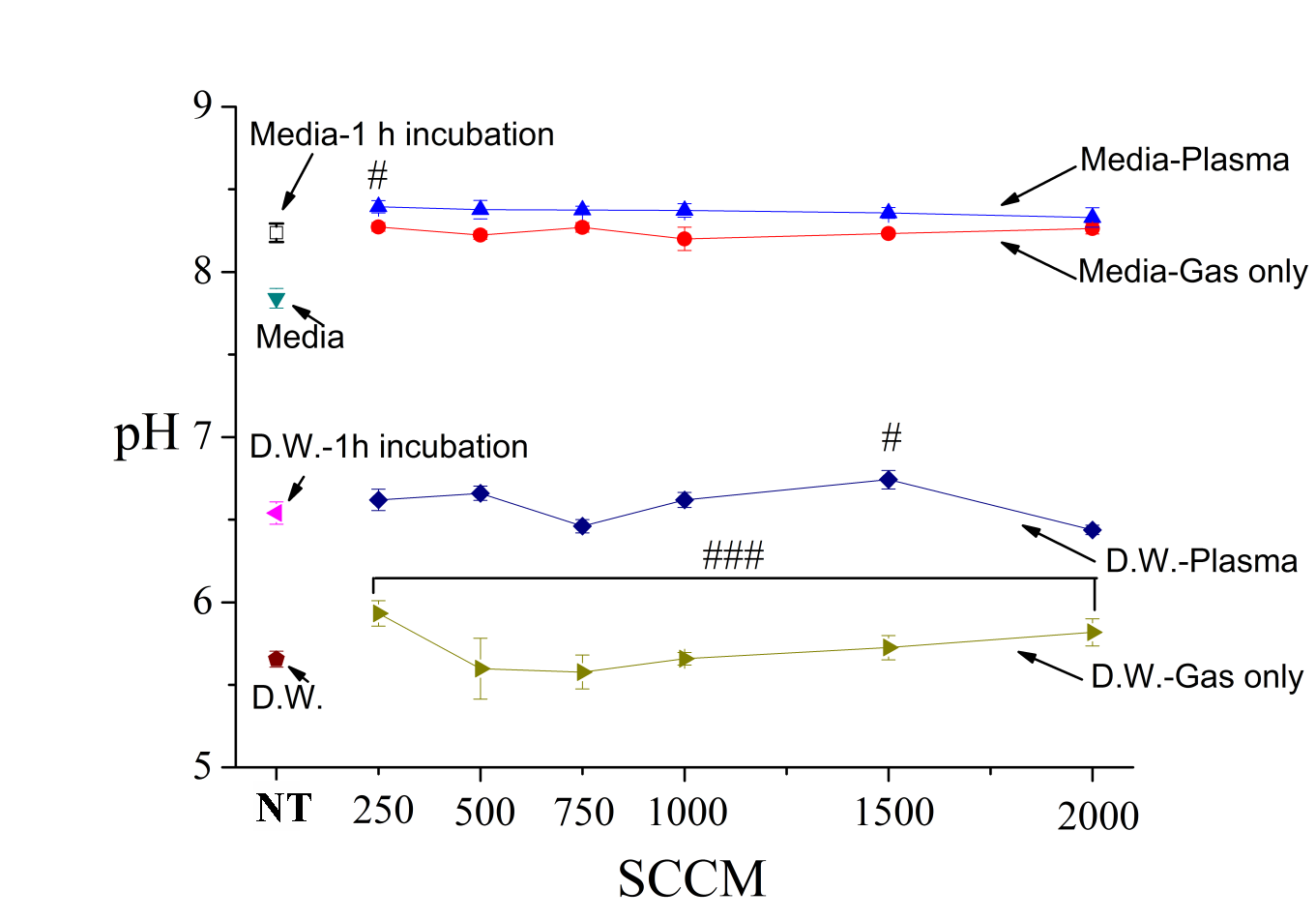

Supplement: S2 Fig — In both liquids, CAP treatment did not show significant difference compared to 1 h incubation media except 250 sccm of media and 1500 sccm of D.W. One # showed significant difference compared to 1 h incubated each solution at a level of 0.05. Three # showed significant difference at a level of 0.001. (TIF) [file pone.0150279.s002.tif]

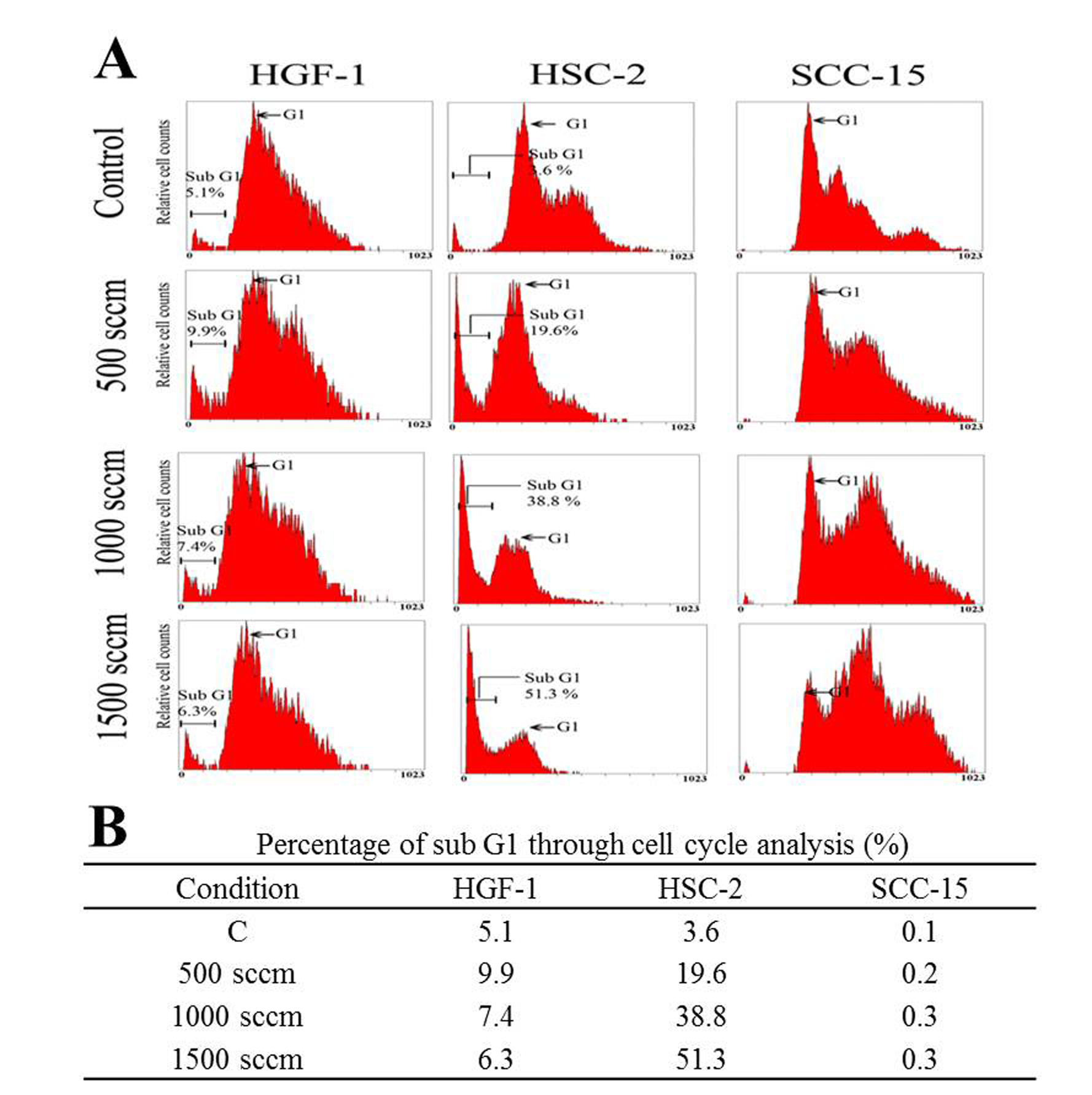

Supplement: S3 Fig — Sub-G1 arrest-related apoptosis was observed only in the HSC-2 cells and depended on the flow rate. (B) Percentage (%) of sub G1 through cell cycle analysis was shown. Assays were performed in triplicate, and representative data were shown. (TIF) [file pone.0150279.s003.tif]
